# Mid-diaphyseal Endosteal Thickening With Subsequent Medullary Narrowing in a Patient With Hallermann-Streiff Syndrome

**DOI:** 10.4021/jocmr704e

**Published:** 2011-11-10

**Authors:** Ali Al Kaissi, Albert Handelbauer, Klaus Klaushofer, Franz Grill

**Affiliations:** a Ludwig Boltzmann Institute of Osteology at the Hanusch Hospital of WGKK and AUVA Trauma Centre Meidling, First Medical Department, Hanusch Hospital, Vienna, Austria; b Orthopaedic Hospital of Speising, Paediatric Department, Vienna, Austria

## Abstract

We report on a 5-year-old girl who presented with the full clinical criteria of Hallermann-Streiff syndrome (HSS). Classically, overtubulation (thin and gracile) bones are the characteristic and constant features in HSS. Interestingly, our present patient manifested unusual mid-diaphyseal endosteal thickening with subsequent medullary narrowing (defective endosteal resorption). To the best of our knowledge no previous reports described such unusual feature in a patient with HSS.

**Keywords:**

Hallermann-Streiff syndrome; Mid-diaphyseal thickening; Radiology

## Introduction

Hallermann-Streiff syndrome (HSS) is a rare congenital anomaly of yet unknown cause, even though it has phenotypic overlap with oculodentodigital dysplasia. The characteristic features are dyscephaly (scaphocephaly or brachycephaly with frontal bossing) and typical face (micrognathia, condylar aplasia, and thin pointed nose), bilateral microphthalmos, and congenital cataract. The negative signs are no anomalies of the ear, no palpebral anomalies, no muscular atrophy, no anomalies of nails, and no or slight mental retardation. The majority of the HSS cases are sporadic. Autosomal dominant inheritance has been suggested but the current data indicates autosomal recessive pattern [1-5].

Classically the long bones of several syndromic associations such as HSS, osteogenesis imperfecta, Marfan syndrome, Cockayne syndrome, Kenny-Caffey syndrome, and osteo-craniostenosis syndrome are principally characterized of being thin and gracile [6,7]. The cardinal bone changes in our current report were derived from conventional radiographic analysis.

## Clinical Report

A 5-year-old girl was referred to the orthopaedic department for clinical evaluation. She was the product of the second pregnancy for a 34-year-old mother and a 40-year-old unrelated father. Clinical examination showed, short stature (below the third percentile) facial characteristics typical of the bird face in HSS. The nose appeared thin, sharp and hooked, the prominence of the chin was absent in lateral the lateral view, microphthlamia and a marked microstomia was evident. In addition, hypotrichosis of the scalp hair, frontal bossing, and dental overcrowding of the upper jaw were evident. Musculoskeletal showed moderate ligamentous hyperlaxity. Hearing, and neurological examinations were normal. She had mild psychomotor retardation and myopia. All other investigations including an abdominal ultrasound, karyotyping, and metabolic tests, which aimed to test calcium, phosphorus, and vitamin D metabolism, were normal. Blood count and alkaline phosphatase were normal as well.

**Figure 1:**
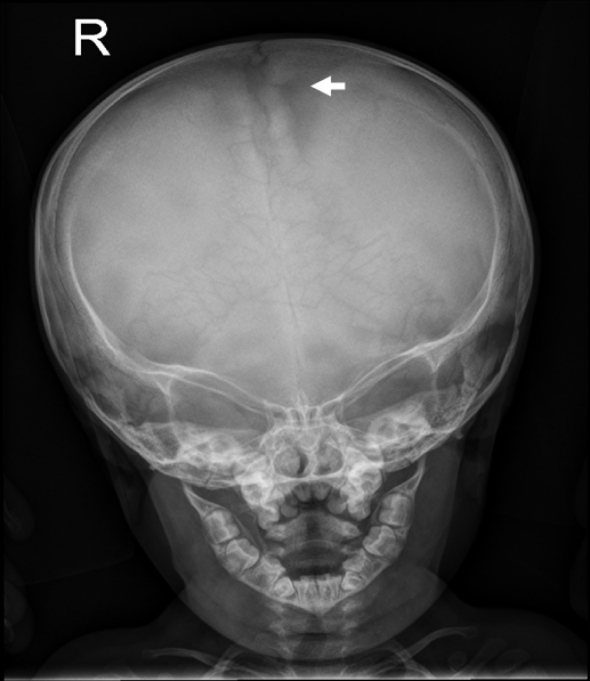
Anteroposterior skull radiograph showed brachycephaly, frontal bossing, defective ossification of the anterior fontanel (arrow), abundant wormian bones parallel to the sagittal suture and dental overcrowding of the upper jaw.

**Figure 2:**
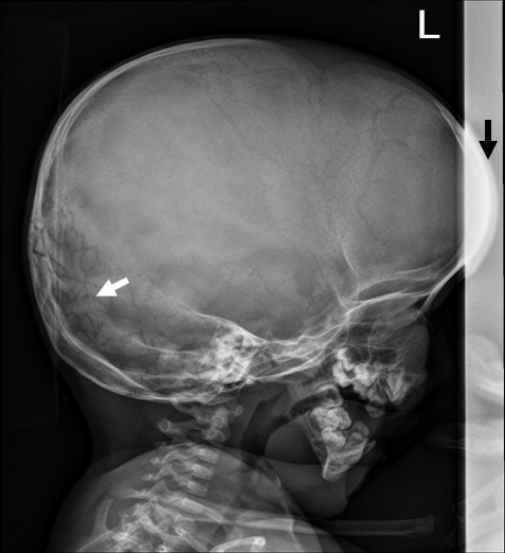
Lateral skull radiograph showed profound frontal bossing (black arrow), thin calvarium, absence of the mandibular angle (temporomandibular joint anomaly with subsequent malocclusion of the teeth), defective ossification of the anterior fontanelle, abundant wormian bones (white arrow) and J-shaped cella turcica.

**Figure 3:**
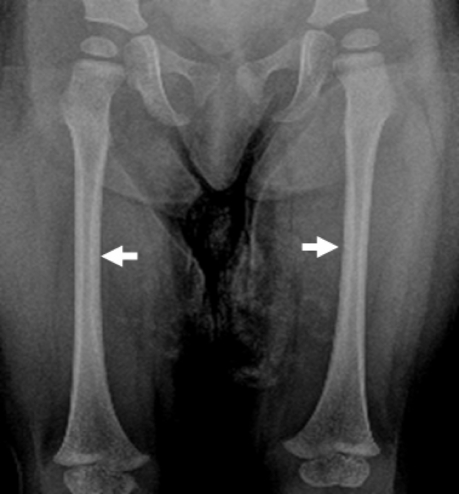
Anteroposterior pelvic-femoral radiograph showed coxa valga associated with mid-diaphyseal endosteal thickening with subsequent medullary narrowing compatible with defective endosteal resorption (arrows).

**Figure 4:**
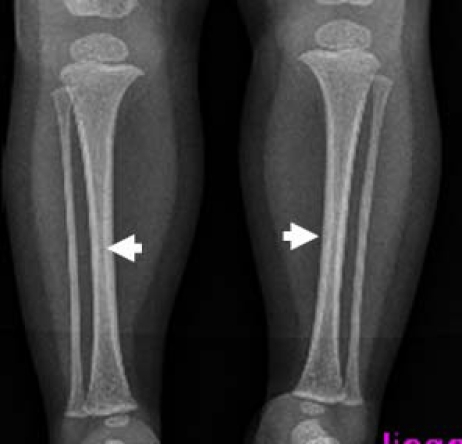
Anteroposterior lower limb radiograph showed relatively broad bones associated with mid-diaphyseal endosteal thickening and medullary narrowing of the tibiae and fibulae respectively (arrows).

On the bases of skeletal survey; Anteroposterior skull radiograph showed brachycephaly, frontal bossing, defective ossification of the anterior fontanel (arrow), abundant wormian bones parallel to the sagittal suture and dental overcrowding of the upper jaw (Fig. 1). Lateral skull radiograph showed profound frontal bossing, thin calvarium, absence of the mandibular angle ( temporo-mandibular joint anomaly with subsequent malocclusion of the teeth), defective ossification of the anterior fontanelle and abundant wormian bones (arrow) and J-shaped cella turcica (Fig. 2). Anteroposterior pelvic-femoral radiograph showed coxa valga associated with mid-diaphyseal endosteal thickening with subsequent medullary narrowing compatible with defective endosteal resorption (arrows) (Fig. 3). Anteroposterior lower limb radiograph showed broad bones associated with mid-diaphyseal endosteal thickening associated with medullary narrowing of the tibiae and fibulae respectively (arrows) (Fig. 4).

## Discussion

Hallermann in 1948 and Streiff in 1950 described patients characterised by ”bird face“, congenital cataract, mandibular hypoplasia, and dental abnormalities. The new syndrome was later defined as HSS, underlining the differences with regard to Franceschetti’s mandibulofacial dysostosis. This condition should be recognized by immediate gestalt. It is the frontal prominence, the thin pointed nose and the small chin which are suggestive, especially in the presence of microphthalmia and cataracts. Slightly later the thinness of the skin around the nose, the forehead, and over the scalp will be noted. The scalp hair remains thin and wispy and the facial features become sharp with age. Other problems include hypotonia. In general the outlook for normal intelligence is good, but mental retardation may occur in about 15% of cases [1-5]. Most cases are single, and Cohen questioned the validity of the few reports of familial cases [8]. He provides an extensive review of 150 cases and suggests that the incidence of the various manifestations is cataract 81-90%, microphthalmia 78-83%, dental anomalies 80-85%, hypotrichosis 80-82%, skin atrophy 68-70% and short stature 45-68%. One of the most severe complications in HSS is respiratory embarrassment and the risk of death due to respiratory complications is not insignificant, particularly during the neonatal period and infancy. Upper airway obstruction may result from small nares and glossoptosis secondary to micrognathia and tracheomalacia. These problems can predispose to obstructive sleep apnea, respiratory insufficiency, pulmonary infection, cor-pulmonale and feeding problems. Endosteal medullary thickening was not a feature.

In normal children the shaft of the long bone, representing the major portion of each long bone, it lies between the metaphyses at either end. In the neonate and young child the diaphysis is extremely vascular with very simple haversian systems. This structure accounts for the pliability of bone with resultant incomplete fractures, rapid rate of healing and excellent capacity to remodel. With age this vascularity of the diaphysis reduces and the bone requires highly complex haversian systems with associated increase in complete fractures and reduced capacity for remodelling. Abnormal configuration of the diaphysis with constriction of its midst part may be affected by various diseases and syndromic entities [9,10]. In children with diaphyseal dysplasia, the appearance consists of smooth cortical thickening, characteristically sparing the epiphyses and metaphyses, with near obliteration of the medullary canal. Radiographs reveal striking diffuse cortical thickening of the diaphyses of the long bones that is characteristically bilateral and symmetrical.

The cortical thickening may be caused either by a disturbance in the periosteal bone formation and apposition or due to a defective endosteal resorption. According to the involved mechanism, the diaphysis may be broadened (increased periosteal apposition), or the medullary cavity may be narrowed (defective endosteal resorption). The prototype of defective endosteal resorption is Van Buchem disease, whereas Camurati-Engelmann disease is a classical example of predominant disturbance of the periosteal apposition [6,11]. Worth and Wollin described a condition of hyperostosis corticalis generalisata which was dominantly inherited. It is radiologically similar to the autosomal recessive disorder of van Buchem syndrome, and some authors refer to the two conditions as endosteal hyperostosis [12].

### Conclusion

We have not been able to elucidate the actual pathogenesis in our patient. There was a noticeable discrepancy between the deficient ossification of the calvaria and the relative endosteal thickening of the long bones. Therefore, it could be possible that our patient might manifest a simultaneous uncoupling of formation and resorption. Finally we wish to stress that assessment of blood count on regular bases is an essential parameter to guard against bone marrow failure.
